# Facial Papulopustular Eruption in an Adult: An Underreported Complication of Miliaria

**DOI:** 10.4269/ajtmh.24-0680

**Published:** 2025-03-13

**Authors:** Akshay Meena, Arun Somasundaram, Sivaranjini Ramassamy

**Affiliations:** Dermatology and STD, Jawaharlal Institute of Postgraduate Medical Education & Research, Pondicherry, India

**Figure 1.**
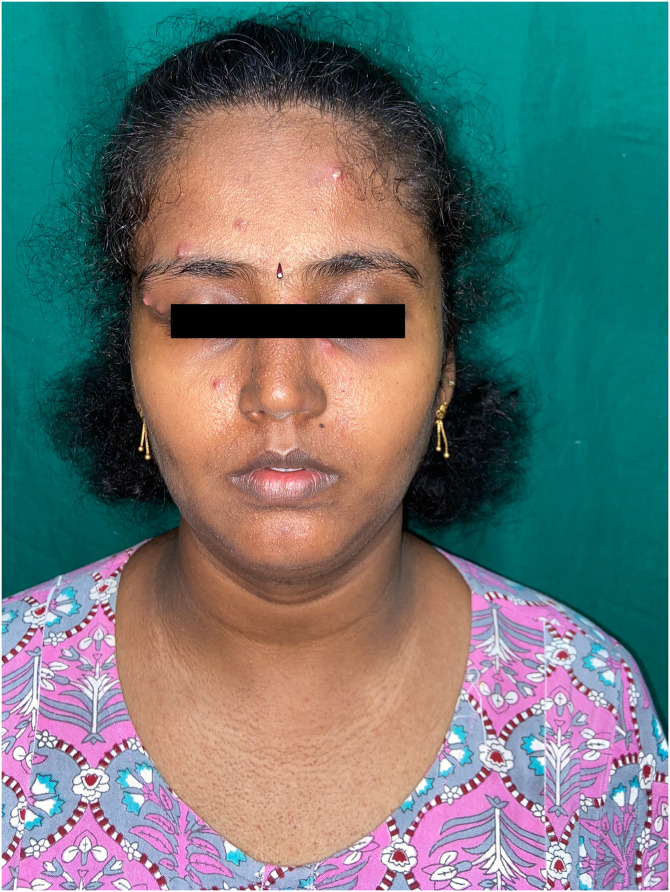
Discrete pin-head pustules on forehead and peri-orbital area with surrounding miliaria on the face and trunk.

A 35-year-old woman presented with asymptomatic pus-filled lesions on her face for 5-days duration. On cutaneous examination, there were multiple discrete, minute, nontender, pinhead-sized pustules predominantly distributed on her forehead and periocular areas. Additionally, there were extensive pinpoint erythematous papules and superficially clear, thin-walled vesicles, 1–2 mm in diameter without surrounding inflammation, evenly distributed on her face and trunk (Figure 1). A swab culture from one of the pustules grew *Staphylococcus aureus*. Based on the clinical findings and laboratory results, a diagnosis of Periporitis staphylogenes with accompanying miliaria was made. The patient was treated with oral Cloxacillin for 5 days, and advised to keep her living environment cool. A follow-up exam after 7 days showed significant improvement in clinical symptoms.

Periporitis staphylogenes is an inflammation occuring around sweat ducts, caused by a superimposed *Staphylococcus aureus* infection. Although most commonly appearing in neonatal skin, Periporitis staphylogenes can occasionally affect adults.^1^ The lesions are typically discrete, minute, pinhead- to pea-sized papules and pustules, which can appear almost anywhere on the skin, but are commonly found on the scalp, forehead, neck, upper chest, shoulders, upper extremities, and back.^2^ The differential diagnoses include folliculitis, furuncles, demodex folliculitis, acne vulgaris, and papulopustular rosacea. *Demodex* folliculitis is characterized by itchy inflammatory papules and pustules on the face and neck, in immunosuppressed individuals with poor hygiene. A skin mount using potassium hydroxide or cyanoacrylate adhesive showing more than five mites per cm^2^, is a clue to the diagnosis. Acne vulgaris presents with asymptomatic polymorphic skin lesions, including: comedones, inflammatory papules, pustules, and nodules that sometimes leave scarring on the face, chest, and back in adolescents. The presence of comedones, folliculocentricity, and a lack of pruritus is more consistent with acne vulgaris than folliculitis. Papulopustular rosacea primarily affects adults and presents with pustules and erythematous papules on the central face with a background of photosensitivity and telangiectasias. Exacerbating factors, such as consumption of alcohol, spicy foods, and sun exposure may be present in patients with rosacea. Periporitis staphylogenes can be clinically distinguished from variants of miliaria such as miliaria pustulosa by characteristically deeper degree of furuncles/folliculitis with lack of tendency to form a central pus-point, local warmth, absence of significant tenderness, and lack of folliculocentricity. If left unnoticed, periporitis can evolve into sweat-gland abscesses. Bacterial cultures from the pustules or abscesses typically yield *Staphylococcus aureus*, confirming the diagnosis, as in our case. The preferred treatment of periporitis staphylogenes is combined antibiotic therapy and miliaria control measures. The presented case highlights this common, underreported complication of miliaria in tropical countries that can occur across all age groups.
